# Brazil's health tax at a crossroads: safeguarding the constitutional victory against ultra-processed foods

**DOI:** 10.1016/j.lana.2025.101363

**Published:** 2026-01-02

**Authors:** Felipe Silva Neves, Larissa Loures Mendes, Eduardo Augusto Fernandes Nilson, Inês Rugani Ribeiro de Castro, Rafael Moreira Claro, Daniela Silva Canella, Ísis Eloah Machado, Ariene Silva do Carmo, Mariana Carvalho de Menezes, Deborah Carvalho Malta

**Affiliations:** a Group for Studies, Research and Practices on Food Environment and Health (GEPPAAS), Department of Nutrition, School of Nursing, Federal University of Minas Gerais (UFMG), Brazil; b Department of Nutrition, Institute of Biological Sciences (ICB), Federal University of Juiz de Fora (UFJF), Brazil; c Department of Nutrition, School of Nursing, Federal University of Minas Gerais (UFMG), Brazil; d Oswaldo Cruz Foundation (FIOCRUZ), Brasília, Brazil; e Rio de Janeiro State University (UERJ), Rio de Janeiro, Brazil; f School of Medicine, Federal University of Ouro Preto (UFOP), Brazil; g School of Nutrition, Federal University of Ouro Preto (UFOP), Brazil; h Department of Maternal and Child Nursing and Public Health, School of Nursing, Federal University of Minas Gerais (UFMG), Brazil

Brazil faces a watershed moment for its public health policy. In 2023, the country achieved a constitutional milestone by instituting a Selective Tax to discourage the consumption of products detrimental to health and the environment.^1^ As Latin America's largest nation, with over 212 million inhabitants, Brazil's health policies exert considerable influence both regionally and globally. Yet this historic advance faces a constant risk of erosion from legislative manoeuvres driven by corporate interests, a conflict that epitomises the global tension between evidence-informed policymaking and the aggressive commercial determinants of health.

The stakes are high. Each year, non-communicable diseases (NCDs) account for over 700,000 deaths in Brazil, representing more than half of all fatalities.^1^ A principal driver of this epidemic is the widespread consumption of ultra-processed foods (UPF), consistently linked to a broad spectrum of adverse health outcomes.^2^ In 2019 alone, an estimated 57,000 premature deaths in Brazil were attributable to UPF.^3^ The economic toll is severe: diseases and deaths associated with these products impose an annual burden of approximately USD 239 million on the public budget, including USD 186.7 million in direct costs to Brazil's Unified Health System (*Sistema Único de Saúde*, SUS).^1^ This crisis also extends to the environment; in Brazil, high-UPF diets have carbon and water footprints 14.4% and 22.8% higher, respectively, than low-UPF diets.^4^ Such data underscore a multidimensional impact that necessitates a robust fiscal response to ensure the long-term sustainability of both the SUS and planetary health.

These challenges are further compounded by the global syndemic of obesity, undernutrition, and climate change, which share common structural drivers. In Brazil, 2023 data indicate that over 61% of individuals aged 18 years and older in state capitals and the Federal District were overweight, and 24% were living with obesity.^5^ Projections suggest that by 2030, 39% of the adult population, representing an estimated 68 million individuals, will be living with obesity. This trajectory reflects a critical systemic failure, as current estimates indicate that no nation is on track to meet the 2030 global targets for halting the rise in obesity.^6^ Dietary patterns play a central role, particularly in contexts of social vulnerability and risk where the commercial determinants of health are more aggressive.^2^^,^^7^

Recent consolidated 2024 data from the Food and Nutrition Surveillance System (*Sistema de Vigilância Alimentar e Nutricional*, SISVAN) reinforce this alarming panorama: more than 80% of children monitored by the Bolsa Família, a conditional cash transfer programme for low-income families, consumed at least one UPF on the preceding day. The most frequent items were sandwich cookies, sugar-sweetened beverages (SSBs), and packaged snacks, illustrating how economic vulnerability and market-driven food environments facilitate the consumption of low-cost, nutrient-poor products. Widespread availability remains a major concern, as SSBs are the most commercialised UPF in private schools and the most prevalent UPF in the vicinity of both public and private schools.^8^

In response, Brazil's tax reform, initiated in 2023, represented a major advance by establishing a national Selective Tax with the explicit extra-fiscal objective of discouraging the consumption of products detrimental to health and the environment. However, this constitutional victory was swiftly compromised during the regulatory phase. Although a broad coalition of experts and civil society organisations advocated for the inclusion of an extensive range of UPF categories, corporate political activity narrowed the final legislation. Consequently, SSBs became the sole UPF class subject to the tax, alongside tobacco, alcoholic beverages, and various non-food goods including vehicles, aircraft, vessels, mineral resources, betting games, and fantasy sports. This decision disregarded technical recommendations which, grounded in robust epidemiological evidence, proposed including UPF such as processed meats, sweetened dairy drinks, and industrialised pastries. Such exclusions create a substantive loophole, incentivising a shift in consumption toward untaxed but equally harmful alternatives, a substitution pattern already underway.^1^^,^^2^ Ultimately, this prioritisation of corporate profit over population health underscores that regulatory integrity is a fundamental requirement for any effective food system transformation.^7^

A recent threat emerged during the regulation of tax rates. A provision was inserted into a bill focused on administrative tax procedures (Complementary Law Bill No. 108/2024) to impose a maximum tax rate of merely 2% on SSBs. This manoeuvre amounted to a calculated attempt to undermine the measure, granting preferential treatment to an industry already benefiting from fiscal incentives estimated at USD 678.6 million annually, primarily via the Manaus Free Trade Zone, a special economic area offering tax exemptions and subsidies to attract industrial investment.^1^ Although this attempt was narrowly defeated following intense mobilisation by civil society organisations and academia, it reflects a familiar playbook of industry interference: first narrow the scope of the measure, then neutralise its effectiveness through symbolic rates.

The rejection of the 2% cap represents a victory, but the priority now shifts to ensuring that final rates are high enough to protect health. International literature demonstrates that only tax rates of at least 20% of the final retail price, as recommended by the World Health Organization, are capable of inducing substantive changes in consumption patterns.^9^ Robust evidence from Mexico, the United Kingdom, and South Africa confirms that taxation at this level leads to measurable and sustained reductions in sales and consumption.^7^ This approach is now a consolidated global strategy, adopted by more than 50 jurisdictions worldwide to address obesity and NCDs. These measures span nations across diverse income brackets, including Chile, France, Hungary, Portugal, and several countries in the Pacific and the Persian Gulf, as well as municipalities in the United States. This global experience reinforces the efficacy of fiscal policy.^1^^,^^7^^,^^9^ By contrast, a 2% limit would be purely symbolic, incapable of generating meaningful price signals and representing a deliberate attempt to hollow out the measure before implementation while obstructing alignment with international best practices.

Local evidence estimates a price elasticity of −1.19 for SSBs, suggesting that a 20% price increase would reduce consumption by nearly 24%.^10^ The stakes are considerable, as diseases associated with these beverages cost the SUS over USD 535.7 million annually. Implementing any rate notably below the 20% threshold, and most critically the attempted 2% limit, would perpetuate an irrational cycle in which the state effectively subsidises an industry while bearing the fiscal and health burdens its products cause. This cycle is already evident with tobacco, a sector where Brazil is a recognised global leader in control policies. Despite this leadership, annual federal tax revenue of approximately USD 1.45 billion is significantly overwhelmed by USD 27.9 billion in annual costs attributable to negative health outcomes, representing 1.6% of the national Gross Domestic Product and providing a stark warning that insufficient tax rates fail to mitigate health-related economic burdens.^1^

Brazil stands at a crossroads. Upholding a substantive tax would consolidate a landmark public health achievement, whereas allowing its dilution would erode the foundations of fiscal health policy and the state's constitutional duty to safeguard health. The world is watching; the decision made in Brazil will set a precedent in the global struggle to prioritise human and planetary well-being over corporate power.

## Declaration of interests

F.S.N. is affiliated with the Group for Studies, Research and Practices on Food Environment and Health (GEPPAAS), Department of Nutrition, School of Nursing, Federal University of Minas Gerais (UFMG), and with the Department of Nutrition, Institute of Biological Sciences, Federal University of Juiz de Fora (UFJF). L.L.M. is affiliated with the Department of Nutrition, School of Nursing, UFMG, and coordinates GEPPAAS. E.A.F.N. is affiliated with the Oswaldo Cruz Foundation (FIOCRUZ), Brasília. I.R.R.C. and D.S.C. are affiliated with the State University of Rio de Janeiro (UERJ), Rio de Janeiro. R.M.C. and M.C.M. are affiliated with the Department of Nutrition, School of Nursing, UFMG, and are members of GEPPAAS. I.E.M. is affiliated with the School of Medicine, Federal University of Ouro Preto (UFOP). A.S.C. is affiliated with the School of Nutrition, UFOP, and is a member of GEPPAAS. D.C.M. is affiliated with the Department of Maternal and Child Nursing and Public Health, School of Nursing, UFMG. All contributions were made in a strictly technical and scientific capacity. The authors declare no competing interests.
